# Supplementary material for: COVID-19: Post-recovery long-term symptoms among patients in Saudi Arabia
Source: PLoS One. 2021 Dec 8;16(12):e0260259. doi: 10.1371/journal.pone.0260259 (PMC8654233; doi:10.1371/journal.pone.0260259)
Supplement: Attachment — Submitted filename: Response to the Reviewers.docx [file pone.0260259.s003.docx]

**Point-by-Point Response to the Reviewers’ Comments**

**Manuscript Number:** PONE-D-21-02645

**Manuscript title:** COVID-19: Post recovery long-term symptoms among patients in Saudi Arabia

**Journal:** PLOS ONE

We are grateful to the reviewers for their thorough reviews and for constructive comments, which enabled us to further improve our manuscript. Concerns raised have been addressed below.

Please note that all the changes in the revised manuscript are highlighted.

**REVIEWERS REPORTS:**

**Review Comments to the Author**

Please use the space provided to explain your answers to the questions above. You may also include additional comments for the author, including concerns about dual publication, research ethics, or publication ethics. (Please upload your review as an attachment if it exceeds 20,000 characters)

**Author’s Response:**

We highly appreciate to the reviewers and the editors for their thorough reviews. The manuscript has been revised exactly in the same ways as per of your suggestions. Please note that all changes in the revised manuscript are highlighted.

**Reviewer 1: Report**

In their paper COVID-19: Post recovery long-term symptoms among patients in Saudi Arabia, Khodeir et al aimed to identify long-term symptoms in different body systems symptoms following COVID-19, their severity, and their duration as a first step in building a system to classify post-recovery long-term symptoms of coronavirus disease 2019 (COVID-19). They conclude that Long-term symptoms after recovery from COVID-19 warrant patient follow-up. The authors propose a classification system as a starting point to guide the identification and follow-up of long-term symptoms post-recovery, and recommend larger-scale studies to broaden the definition of recovery from COVID-19, which appears to have two phases, acute and chronic. While the goal of this paper is interesting, several improvements can be made to this paper.

**Author’s Response:**

We highly appreciate for your excellent review, which definitely helped us to further improve the quality of the manuscript. All suggestions have now been incorporated in the revised manuscript.

**Specific Comments**

**Comment # 1:**

The methods section requires expansion, a copy of the questionnaire, its translation etc. needs to be placed in the methods.

**Author’s Response:**

We highly appreciate you for your thorough review. As suggested, the used questionnaire and its translation have now been included in the methods subsection ‘Instrument development and measures’ on page 5 and 6 and the complete details of used questionnaire with translation have now been added in table 1 of the revised manuscript on page 7 and 8.

**Comment # 2.**

The statistics portion requires more sophisticated analysis. For example, usually the mean is reported with SD, and the median is reported with the interquartile range and/or range. Here the authors report the mean SD and range. Also, to develop a score or classification system, there has to be an end point. For example, what is the goal of this classification? Is there a stage? Is there a score? Is one type better than the others. Several adjustments of baseline characteristics are needed. Saudi versus non-Saudi is not needed, Race, ethnicity, work, etc. may be better. LOTS of improvement may be made to this section.

**Author’s Response:**

We highly appreciate for your excellent review and for pointing out these. As suggested, a detailed statistical analysis have now been performed and included in the methods section, results section and also in the discussion sections. Specifically, we have now been added more detailed scoring system with different levels of priorities for patient follow up and cut off values. Please see the newly statistical data in the revised Table 3, 4, 5 and 6 on page 12,13,19-21, respectively and also see the newly added figure 1 and 2. Furthermore, the data on nationality of the studied subject have now been omitted as suggested. Please note table numbers in the revised manuscript are changed.

**Comment # 3.**

The results section needs more details. I think reporting the “classification system” reposting a “scale for it” briefly reporting the populations. Again, improvements need to be made

**Author’s Response:**

Thanks for your useful suggestion which definitely helped us to further improve the quality of the manuscript. As suggested, more details in the results section have now been included in the revised manuscript. Please see the highlighted text in Tables 2-4 on page 11-13 and also see the added data in figures 1-3. Furthermore, please also see the highlighted text in the results section on page 17, line 3-23 and page 18, line 1-4.

**Comment # 4.**

The tables could become one table

**Author’s Response:**

Thanks. All possible modifications in the tabular presentation have been made in the revised manuscript. In addition, the data have also now been presented in the forms of figures. Please note table numbers in the revised manuscript are changed.

**Comment # 5.**

Figures would help deliver the message better

**Author’s Response:**

Thanks. As suggested, figures have now been included in the revised manuscript. Please see the data in newly data figures 1-3.

**Comment # 6.**

The conclusion is too long, and should not have references or references to tables, this should move to the discussion. The conclusion summarizes your findings, not others.

**Author’s Response:**

As suggested, the conclusion section has now been revised and the word count has now been significantly reduced. All text with citations has been shifted and discussed in the discussion section. Please see the highlighted text on page 17, line 3-23, page 18, line 1-4 in the discussion section and also page 21, line 10-20 in the conclusion section of the revised manuscript.

**Comment # 7.**

In summary, while the findings and goal of the study is quite good, the structure of this paper could improve.

**Author’s Response:**

We highly appreciate your for your constructive criticisms and positive comments, which definitely have improved the quality of the manuscript.

**Reviewer 2: Report**

In the manuscript authored by Mostafa Khodeir et al, the authors reported results obtained from an on-line survey about the post recovery long-term symptoms among patients in Saudi Arabia. Despite manuscript is valuable in the attempt here below I reported my comments and suggestions.

**Author’s Response:**

We are grateful to you for your thorough review and for your constructive comments. All concerns raised have been addressed below. Please note that all major changes in the revised manuscript are highlighted.

**Comment # 1**:

It is not clear in the method section how subjects have been included. Authors stated: "The estimated sample size was 663, which was obtained by statistical calculation from the official Saudi Ministry of Health website, which announced the number of recovered cases with a 99% confidence level and a 5% margin of error". Thus, why authors did not contacted directly those 663 persons? It is correct the 99% confidence level?

**Author’s Response:**

Thanks for your thorough review and pointing out these. As suggested, more details in the methods section have now been provided to clarify how the participants were included. Now we have changed the confidence level to 95% and the details of recalculation of the sample size have been provided on page 9, line 1-4 in the revised manuscript. We are also pleased to inform that our survey response exceeded the calculated sample size in both situations of CI 95% or 99%. We would also like to inform that we had not contacted the cases directly in person because of some obstacles such as (a) social and precautionary measures and restrictions in the country, (b) fear of recovered persons to contact hospital or clinics for reinfection (still deficient information about the disease), (c) difficulties in transportation and feasibility time of the respondents, (d) there is still no clear concept about the need of recovered COVID-19 cases for follow up, therefore we considered the piloted sample (40 cases) to continue the study by online survey not to bother the respondents and also the urgency to know more about the disease to recommend all required measures to manage COVID-19 especially those missed and considered recovered by just recovery from acute symptoms. Now we have clearly mentioned that the survey was conducted online and the limitations of the study have also been clearly defined in the revised methods section on page 6, line 15-22.

**Comment # 2:**

Thus it is not clear if the 979 subjects who performed the on-line survey were all recovered for COVID-19.

**Author’s Response:**

Yes. All were recovered from acute symptoms of COVID-19. We have now added this information in the results section on page 9, line 17 and 18 of the revised manuscript.

**Comment # 3:**

The group analyzed is mainly composed from young adults since only 2.7% were >50 years. Thus results obtained are mainly representative of the post recovery in young adults patients.

**Author’s Response:**

Yes, this information is 100% correct. This could be explained as the survey being done online which is easier for young. This important information has now been added in the limitations of the study of the revised manuscript.

**Comment # 4:**

No statistical analysis is present. Only mean and frequency.

**Authors Response:**

As suggested, more statistical analysis details have now been included throughout the manuscript including tables and figures. And the details of the used statistical methods have been summarized in the revised manuscript on page 9, line 6-14.

**Comment # 5:**

Again, authors should also mentioned that on-line survey can be affected from the subjective perception of the interviewed.

**Author’s Response:**

We highly appreciate for your thorough review and pointing out this. As suggested, this detail has now been included in the limitations of the study on page 16&17, and also in the limitation of questionnaire on page 6 of the revised manuscript.

**Comment # 6:**

Elderlies are highly affected with harmful post recovery long-term symptoms, but this group is missing.

**Author’s Response:**

Thanks for your suggestion and pointing out this. As suggested, the detail of this missing age group has now been included. Now we performed analysis on the basis of age group distribution where elderlies were characterized as 60 or above years of age. These details are summarized in Table 2 of the revised manuscript on page 11.

**Comment # 7:**

In tables included in the manuscript, when authors mention: "mean" and "frequency" please include also to what they are refering for, **as days**.

**Author’s Response:**

Thanks. As suggested, the tables have now been revised and the suggested details of referring of days have now been included in the revised tables 3 and 5 on page 12&13 and their related text has now been added and highlighted in the results section on page 9 and 10 and discussed in the discussion section on page 12-13 in the revised manuscript.

**Reviewer's Responses to the Editorial Questions: Author’s Response**

**Comment # 1:** Is the manuscript technically sound, and do the data support the conclusions?

The manuscript must describe a technically sound piece of scientific research with data that supports the conclusions. Experiments must have been conducted rigorously, with appropriate controls, replication, and sample sizes. The conclusions must be drawn appropriately based on the data presented.

**Reviewer #1**: Partly

**Reviewer #2:** Partly

**Author’s Response:**

We highly appreciate both of the reviewers for their thorough review and constructive criticisms, which helped us to further improved the quality of the manuscript. We have addressed all the concerns raised above and hope that the both of you find the answers satisfactory.

**Comment # 2:** Has the statistical analysis been performed appropriately and rigorously?

**Reviewer #1:** No

**Reviewer #2:** No

**Author’s Response:**

Thanks. This part has also been significantly improved by addition of more statistical analyzed data. Please see the highlighted text in the methods section, results section and particularly tables and figures of the revised manuscript.

**Comment # 3:** Have the authors made all data underlying the findings in their manuscript fully available?
The [PLOS Data policy](http://www.plosone.org/static/policies.action#sharing) requires authors to make all data underlying the findings described in their manuscript fully available without restriction, with rare exception (please refer to the Data Availability Statement in the manuscript PDF file). The data should be provided as part of the manuscript or its supporting information, or deposited to a public repository. For example, in addition to summary statistics, the data points behind means, medians and variance measures should be available. If there are restrictions on publicly sharing data—e.g. participant privacy or use of data from a third party—those must be specified.

**Reviewer #1:** Yes

**Reviewer #2:** Yes

**Author’s Response:**

We highly appreciate for the positive comments.

**Comment # 4:** Is the manuscript presented in an intelligible fashion and written in standard English?
PLOS ONE does not copyedit accepted manuscripts, so the language in submitted articles must be clear, correct, and unambiguous. Any typographical or grammatical errors should be corrected at revision, so please note any specific errors here.

**Reviewer #1**: No

**Reviewer #2**: Yes

**Author’s Response:**

Thanks. As suggested by the editorial members, the manuscript has been edited by an expert English Editing Company (Editage) and also has now been revised by one of our English language experts. Now we hope that the revised manuscript will now meet the high standards of the journal and suitable for publication in *PLOS ONE*.

# EDITORIAL COMMENTS

**Comment # 1:**

Please ensure that your manuscript meets PLOS ONE's style requirements, including those for file naming. The PLOS ONE style templates can be found at

<https://journals.plos.org/plosone/s/file?id=wjVg/PLOSOne_formatting_sample_main_body.pdf>

and

https://journals.plos.org/plosone/s/file?id=ba62/PLOSOne_formatting_sample_title_authors_affiliations.pdf

**Author’s Response:**

We highly appreciate the editorial board of PLOS ONE for giving us an opportunity to revise our manuscript. The manuscript has now been revised exactly in the same format as instructed.

**Comment # 2:**

Please provide additional details regarding participant consent. In the ethics statement in the Methods and online submission information, please ensure that you have specified what type you obtained (for instance, written or verbal, and if verbal, how it was documented and witnessed). If your study included minors, state whether you obtained consent from parents or guardians. If the need for consent was waived by the ethics committee, please include this information.

**Author’s Response:**

Thanks. Informed consent statement has now been well defined and included in the methods section on page 4, line 24; page 5, lines 1 and line 5-8 in the revised manuscript. Please note all changes made in the revised manuscript are highlighted.

**Comment # 3:**

Please include additional information regarding the survey or questionnaire used in the study and ensure that you have provided sufficient details that others could replicate the analyses. For instance, if you developed a questionnaire as part of this study and it is not under a copyright more restrictive than CC-BY, please include a copy, in both the original language and English, as Supporting Information. Moreover, please include more details on how the questionnaire was pre-tested, and whether it was validated.

**Author’s Response:**

As suggested by you and one of the reviewers, the complete details of the questionnaire have now been included and its copy with Arabic translation has been inserted in the methods section on page 7 and 8. Furthermore, all possible details of the questionnaire including its validation have now been added in the methods subsection ‘Instrument development and measures’ on page 5 and 6 of the revised manuscript.

**Comment # 4:**

Thank you for stating the following financial disclosure: "The funders had no role in study design, data collection and analysis, decision to publish, or preparation of the manuscript." At this time, please address the following queries:

**Comment # 4 (a):** Please clarify the sources of funding (financial or material support) for your study. List the grants or organizations that supported your study, including funding received from your institution.

**Author’s Response:**

We as authors hereby declared that this study has no association with any of the funding agencies.

**Comment # 4 (b):** State what role the funders took in the study. If the funders had no role in your study, please state: “The funders had no role in study design, data collection and analysis, decision to publish, or preparation of the manuscript.”

**Author’s Response:**

We declared that “The funders had no role in study design, data collection and analysis, decision to publish, or preparation of the manuscript”. This statement has also now been mentioned in the ‘Funding support’ section of the revised manuscript on page 22, line 14-16.

**Comment # 4 (c):** If any authors received a salary from any of your funders, please state which authors and which funders.

**Author’s Response:**

None.

**Comment # 4 (d):** If you did not receive any funding for this study, please state: “The authors received no specific funding for this work.” Please include your amended statements within your cover letter; we will change the online submission form on your behalf

**Authors Response:**

Thanks. We hereby declared that “The authors received no specific funding for this work.”

**Comment # 5:**

Please include your tables as part of your main manuscript and remove the individual files. Please note that supplementary tables (should remain/ be uploaded) as separate "supporting information" files

**Authors Response:**

Thanks. As suggested, all tables have now been inserted in the main manuscript.

**Comment # 6:**

Additional Editor Comments (if provided):

Dear Authors, thank you for submitting in Plos One. Your manuscript has been assessed by relevant experts from the field. They found manuscript interesting but raised some concerns in methodology (study instrument validation and translation, sampling technique and methods of recruitment) and interpretation of results. It is requested to please consider the comments of reviewers.

**Author’s Response:**

Thank you for giving us the opportunity to revise this manuscript. The manuscript has been revised in light of the reviewers’ and editorial comments for publication and we believe that the revised manuscript will now meet the high standards of the journal and suitable for publication in “PLOS ONE”.

**Corresponding Author**

**Zafar Rasheed,** MS, PhD, PGDCA.

ORCID ID: <http://orcid.org/0000-0002-8651-4218>
