# Supplementary material for: COVID-19: Post-recovery long-term symptoms among patients in Saudi Arabia
Source: PLoS One. 2021 Dec 8;16(12):e0260259. doi: 10.1371/journal.pone.0260259 (PMC8654233; doi:10.1371/journal.pone.0260259)
Supplement: Attachment — Submitted filename: Re-Response to the Reviewers.docx [file pone.0260259.s004.docx]

**Point-by-Point Re-Response to the Reviewers’ Comments**

**Manuscript Number:** PONE-D-21-02645R1

**Manuscript title:** COVID-19: Post recovery long-term symptoms among patients in Saudi Arabia

**Journal:** PLOS ONE

We are grateful to the reviewers and editors for their thorough reviews and for constructive comments, which enabled us to further re-improve our manuscript. Concerns raised have been re-addressed below.

Please note that all major changes in the Re-revised manuscript are highlighted.

**REVIEWERS' COMMENTS:**

**Reviewer's Responses to Questions**

## Comments to the Author

---------------------------------------------------------------------------------------------------------------------
1. If the authors have adequately addressed your comments raised in a previous round of review and you feel that this manuscript is now acceptable for publication, you may indicate that here to bypass the “Comments to the Author” section, enter your conflict of interest statement in the “Confidential to Editor” section, and submit your "Accept" recommendation.

**Reviewer # 2:** All comments have been addressed

**Author’s Response:** We highly appreciate your positive feedback.

**Reviewer # 3:** All comments have been addressed

**Author’s Response:** We highly appreciate your positive feedback.

**Reviewer # 4:** (No Response)

**Author’s Response:** We highly appreciate your thorough review, which helped us to further improve the quality of the manuscript.

**Reviewer # 5:** (No Response)

**Author’s Response:** We highly appreciate your thorough review, which helped us to further improve the quality of the manuscript.

---------------------------------------------------------------------------------------------------------------------

2. Is the manuscript technically sound, and do the data support the conclusions?

The manuscript must describe a technically sound piece of scientific research with data that supports the conclusions. Experiments must have been conducted rigorously, with appropriate controls, replication, and sample sizes. The conclusions must be drawn appropriately based on the data presented.

**Reviewer # 2:** Yes

**Author’s Response:** We highly appreciate your positive feedback.

**Reviewer # 3:** Partly

**Author’s Response:** We highly appreciate for the positive response. The manuscript has now been re-revised in light of your comments.

**Reviewer # 4**: Yes

**Author’s Response:** We highly appreciate your positive feedback.

**Reviewer # 5**: Partly

**Author’s Response:** We highly appreciate your positive feedback. The manuscript has now been re-revised in light of your comments.

---------------------------------------------------------------------------------------------------------------------

3. Has the statistical analysis been performed appropriately and rigorously?

**Reviewer # 2:** Yes

**Author’s Response:** We highly appreciate your positive feedback.

**Reviewer # 3:** Yes

**Author’s Response:** We highly appreciate your positive feedback.

**Reviewer # 4:** Yes

**Author’s Response:** We highly appreciate your positive response.

**Reviewer # 5:** Yes

**Author’s Response:** We highly appreciate your positive response.

---------------------------------------------------------------------------------------------------------------------

4. Have the authors made all data underlying the findings in their manuscript fully available?

The [PLOS Data policy](http://www.plosone.org/static/policies.action#sharing) requires authors to make all data underlying the findings described in their manuscript fully available without restriction, with rare exception (please refer to the Data Availability Statement in the manuscript PDF file). The data should be provided as part of the manuscript or its supporting information, or deposited to a public repository. For example, in addition to summary statistics, the data points behind means, medians and variance measures should be available. If there are restrictions on publicly sharing data—e.g. participant privacy or use of data from a third party—those must be specified.

**Reviewer # 2:** Yes

**Author’s Response:** We highly appreciate your positive feedback.

**Reviewer # 3:** Yes

**Author’s Response:** We highly appreciate your positive feedback.

**Reviewer # 4:** Yes

**Author’s Response:** We highly appreciate your positive feedback.

**Reviewer # 5:** No

**Author’s Response:** We highly appreciate your thorough reviews. The manuscript has now been re-revised as per of your suggestions.

---------------------------------------------------------------------------------------------------------------------

5. Is the manuscript presented in an intelligible fashion and written in standard English?

PLOS ONE does not copyedit accepted manuscripts, so the language in submitted articles must be clear, correct, and unambiguous. Any typographical or grammatical errors should be corrected at revision, so please note any specific errors here.

**Reviewer # 2**: Yes

**Author’s Response:** We highly appreciate your positive feedback.

**Reviewer # 3:** No

**Author’s Response:** We highly appreciate your thorough reviews. The manuscript has now been re-revised as per of your suggestions.

**Reviewer # 4:** Yes

**Author’s Response:** We highly appreciate your positive feedback.

**Reviewer # 5:** Yes

**Author’s Response:** We highly appreciate your positive feedback.

---------------------------------------------------------------------------------------------------------------------

6. Review Comments to the Author

Please use the space provided to explain your answers to the questions above. You may also include additional comments for the author, including concerns about dual publication, research ethics, or publication ethics. (Please upload your review as an attachment if it exceeds 20,000 characters)

**Reviewer # 2:** Authors provided most of the suggestions and modification requested. Now the manuscript has been improved, thus I suggest it for a publication in PLOSone

**Author’s Response:** Many thanks for your positive response.

**Reviewer # 3:** Appreciating the authors for their response to the required comments. However, the manuscript still needs English language editing.

**Author’s Response:** We highly appreciate your positive feedback. This manuscript has been edited by an expert English Editing Company Editage and also has now been Re-revised by one of our English language experts.

**Reviewer # 4:** COVID-19: Post recovery long-term symptoms among 2 patients in Saudi Arabia.

Review report:

**(a)** Shorten the paper to around 6000 words.

**Author’s Response:** As suggested, the manuscript has now been reduced to ~5500 words.

**(b)** Questionnaire need not be shown in the manuscript.

**Author’s Response:** As suggested, the questionnaire has now been omitted from the re-revised manuscript. Respectfully, we would like to inform that we have placed the questionnaire in the previous version of this manuscript as per instructions of reviewer # 1, which has now been presented as a supplementary file.

(c) Rewrite the manuscript with all results in one section.

**Author’s Response:** Thanks. All results have now been presented in one section.

**Reviewer # 5:** With this ongoing World War against this COVID-19 thing, this manuscript addresses a hot topic in which any effort in this concern is highly appreciated. The study is generally scientifically sound however; some important points need to be clarified. These include (on the revised version of the submission):

**1)** Page 5, lines 1-2: The authors wrote: "A survey was administered between September and October 2020 using Google Forms and Twitter as a forum". Well, the conclusions based on the answers extracted from a survey on the social media platform are usually taken with extreme caution. So, this point has to be CLEARLY mentioned in the Limitation(s) Section.

**Author’s Response:** We highly appreciate your positive feedback. As suggested, the limitation section has now been modified by taking caution. The ways of questionnaire administration have now been well discussed in the limitations of the study on page 18, line 12-21 in the re-revised manuscript.

**2)** Page 6, lines 1-2: The authors wrote "The results of the pilot study were not included in any subsequent research". OK, the result of validity and reliability test of the questionnaire has to be included.

**Author’s Response:** Excellent suggestion. As suggested, the validity and reliability test of the questionnaire have now been included in the revised manuscript on page 6, line 1-9 and their associated references have also been cited. Please see the newly added references # 36 and 37 in the reference list on page 24.

**3)** Page 9, line 1: OK, here the readers are definitely going to get confused about how the author managed to get this particular figure (384) as a sample size beside, line 17 on the same page 9 tells a completely different story about the number of subjects recruited (979) with no clue whatsoever. So the authors need to mention the whole story of the sample size calculation.

**Author’s Response:** Thanks for your excellent suggestion. As suggested, this useful information of sample size has now been added in the revised manuscript on page 7, line 4-9.

**4)** Page 16; Line 23: The authors wrote: “This study has few limitations such as”.
OK here is the thing; I encourage the authors to avoid this kind of open statements which include “such as and/or etc.” So, I do urge the authors to precisely count down all these limitations. Speaking of limitation, I suggest gathering all these limitations under one separate section (subtitle).

**Author’s Response:** Thanks. As suggested, all limitations have now been gathered and discussed in the last part of the discussion section on page 18, line 12-21 in the revised manuscript.

**5)** Tables 4 to 6B: (N) is to be mentioned.

**Author’s Response:** Thanks. All tables and figures have now been cited in the results section of the re-revised manuscript.

---------------------------------------------------------------------------------------------------------------------

7. PLOS authors have the option to publish the peer review history of their article (what does this mean?). If published, this will include your full peer review and any attached files.

If you choose “no”, your identity will remain anonymous but your review may still be made public.

Do you want your identity to be public for this peer review? For information about this choice, including consent withdrawal, please see our Privacy Policy.

Reviewer #2: No

Reviewer #3: Yes: Walid Kamal Abdelbasset

Reviewer #4: Yes: Asharaf Abdul Salam

Reviewer #5: No

**Author’s Response:** We highly appreciate all the reviewers for their excellent review, which have improved the quality of the manuscript.

We believe that the Re-revised manuscript will now meet the high standards of the journal and suitable for publication in “PLOS ONE”.

**Corresponding Author**

**Zafar Rasheed,** MS, PhD, PGDCA.

ORCID ID: <http://orcid.org/0000-0002-8651-4218>
