# Supplementary material for: COVID-19: Post-recovery long-term symptoms among patients in Saudi Arabia
Source: PLoS One. 2021 Dec 8;16(12):e0260259. doi: 10.1371/journal.pone.0260259 (PMC8654233; doi:10.1371/journal.pone.0260259)
Supplement: Attachment — Submitted filename: Response to the Reviewers Oct 8, 2021.docx [file pone.0260259.s005.docx]

**Point-by-Point Re-Response to the Reviewers’ Comments**

**Manuscript Number:** PONE-D-21-02645R2

**Manuscript title:** COVID-19: Post recovery long-term symptoms among patients in Saudi Arabia

**Journal:** PLOS ONE

We are grateful to all the reviewers for their thorough reviews and for constructive comments, which enabled us to further re-improve our manuscript. Concerns raised have been re-addressed below.

Please note that all major changes in the Re-revised manuscript are highlighted.

**REVIEWERS' COMMENTS:**

**Reviewer # 1:** Final Report Outcome

**Author’s Response:** We highly appreciate you for the positive feedback

**Reviewer # 2:** Final Report Outcome

**Author’s Response:** We highly appreciate you for the positive feedback

**Reviewer # 3:** Final Report Outcome

**Author’s Response:** We highly appreciate you for the positive feedback

**Reviewer # 4:** Report

COVID-19: Post recovery long-term symptoms among patients in Saudi Arabia

**Comment 1:**

A total of 979 patients 10 recovered from COVID-19 in Saudi Arabia in the study period, of whom 53% were male and 47% were female. Sample size is appreciated as such studies from Saudi Arabia are rare, but not this male-female proportions. It is stated that males are affected more than females. Here the difference is not large.

**Author’s Response:**

It has now been corrected on page 8, line 3 in the re-revised manuscript.

**Comment 2:**

3–5 min (P5; L8) may change to 3-5 minutes

**Author’s Response:**

As suggested, it has now been changed.

**Comment 3:** Instrument development and measures section is one paragraph. Consider splitting into two or even three.

**Author’s Response:**

As suggested, this paragraph has now been splitted into two subsections on page 5 and 6 in the re-revised manuscript.

**Comment 4:** Final questionnaire was structured into 6 sections 10 addressing specific symptoms (P6 L 9-10) – In case the authors adopted any classification system, the same may be referred.

**Author’s Response:**

We mentioned that ‘the questionnaire used in this study was created using a combination of published literature and the literature used was well cited on page 5, line 14 and 15.

**Comment 5:** In P7 the following statements need explanations a. a total of 992 subjects were approached b. 13 subjects were omitted as they were made invalid selection and c. 979 subjects were recruited. Whether used a sample frame or a followed a list of subjects. Sample representativeness needs to be explained.

**Author’s Response:**

Thanks. Sample representativeness has now been mentioned and highlighted on page 7, line 8 in the re-revised manuscript. All subjects were Saudi citizens.

**Comment 6:** Of the 979 respondents’ patients (P8)- use of ‘

**Author’s Response:** It has now been corrected on page 8, line 2.

**Comment 7:** respectively) (Table 3) (P8; L18) – Avoid two parentheses together

**Author’s Response:** Thanks, this has also now corrected.

**Comment 8:** Furthermore, we also found 21 a statistically significant relation of age with the presence of post-recovery COVID-19 long term 22 symptoms degree of severity and/or persistence, such as weakness degree (p= 0.003), persistence (p=0.001), lack of appetite degree (p=0.02), persistence (p=0.003), Insomnia degree (p=0.01), 9 1 loss of smell degree (p=0.002), loss of taste degree and Headache degree (p=0.04 each), cough 2 degree (p=0.01) significant correlation was found between age and persistence of symptoms as 3 fatigue (p=0.004), joint pains (p=0.01), mood changes (p=0.03), nausea (p=0.002) and 4 abdominal pain (p=0.02)( Table 2&3) – Please check the tables and clear confusions by including age in the table titles and columns.

**Author’s Response:**

We have now re-checked both of these tables and the mean (±SD) age of the studied subjects in years has now been added in their titles in the re-revised manuscript as per of your suggestions.

**Comment 9:** The proposed scoring system in this study can be delivered online for post discharge follow-up, and the score of each case can be automatically calculated. The case that will reach the proposed score should be invited for follow up in the clinic, keeping in mind high concerns groups (Table 4 level 1A&B) are first priority follow up group, while lower concerns groups (Table 5 level 2 A&B) are of second priority follow up. The cases not reached the proposed score no need for clinic follow up and should be reassured as mostly their symptoms be self-limited. By this scoring system we can easily pickup patients of concern to be followed up and also decrease load on health services as much as possible. Why these lines stand different????

**Author’s Response:**

This paragraph is linked to our findings. For more clarity to the readers to understand, we have made some modifications in this paragraph on page 7, line 5-15 in the re-revised manuscript.

**Comment 10:** Table 2 shows a wide range days (upto 120 days). It would be nice to explain column by column. You may use either mean days or median days. It is nice to state the statistical test performed for reaching out the p value. Results need to be further elaborated for readers to understand. Use smaller paragraphs in discussions. I mean, break ideas into paragraphs.

**Author’s Response:**

Thanks. The data in mean days as well as median days have already mentioned in the Table 2. As suggested, the statistical test used to measure p values has now been added in the table legend. Moreover, the discussion sections has now been divided into smaller paragraphs. Please note only major changes in the re-revised manuscript are highlighted.

**Comment 11:** we can’t expect who will suffer (P17, L 9) may be reworded. Our scoring system will help to broaden the view of the scoring system that used to classify the acute cases of COVID-19 into mild, moderate, severe, or critical which proposed by The Chinese National Health Commission [64]. We aimed by our scoring system to map and score, as an initial step to build a scoring system, the recorded long-term symptoms to avoid missing such cases who may suffer a sequalae later on, keeping in mind the exact pathogenesis is still unclear. Our proposed scoring system can be delivered online for post discharge follow-up, and can be automatically calculated the score of each case. The case that will reach the proposed score (score 2) should invited for follow up in the clinic, keeping in mind high concerns groups are first priority follow up group, while lower concerns groups are of second priority follow up. The cases not reached the proposed score no need for clinic follow up and should be reassured as mostly their symptoms be self-limited. By this scoring system we can easily pickup patients of concern to be followed up and also decrease load on health services as much as possible. Again, this scoring system could be applied online weekly and so we can get a wider scale and broader view on behavior of these long-term symptoms. Our proposed scoring system and categorization of patients into high concern and lower concern groups may considered as an initial step that help and encourage a wider scale studies in different countries to confirm and refine the findings by considering geographical distribution and a larger number of COVID-19 cases. This will help to identify priorities in follow-up among patients according to longer-term symptoms and to avoid prolonged suffering in those who recover from COVID-19. (P17-18). Do not match with the discussions, that is wrongly placed.

**Author’s Response:**

Thanks. This paragraph has now been omitted from the discussion section of the re-revised manuscript.

**Comment 12:** Acknowledgments: The authors thank all health services personnel and volunteers who are on the front lines of the pandemic and have expended great effort to fight the disease all over the world. Special thanks to those who sacrificed their lives to save thousands of others. The authors also thank those who devoted their time and exerted extraordinary effort to create vaccines to ease—if not eliminate— 22 suffering around the world. Authors may acknowledge those who helped them in this research and manuscript preparation.

**Author’s Response:**

The paragraph you copied from the acknowledgement section and pasted above has already mentioned the acknowledgments of all the services, personnel and volunteers that we utilized in the preparation of this manuscript. Respectfully, we are very pleased to inform that all authors themselves participated in this research and in the preparation of manuscript.

### Reviewer # 5: Report

Because they are too many and significantly affect the reliability of all the conclusions and recommendations drawn, all limitations are to be gathered in a separate section under the subtitle "LIMITATIONS" and NOT as a paragraph in the "Discussion" section.

**Author’s Response:**

Thanks for your review and for the suggestions. As suggested, study limitations have now been presented in a separate section.

We believe that this revised manuscript will now meet the high standards of the journal and suitable for publication in “PLOS ONE”.

**Corresponding Author**

**Zafar Rasheed,** MS, PhD, PGDCA.

ORCID ID: <http://orcid.org/0000-0002-8651-4218>
